# αEβ7 Integrin Identifies Subsets of Pro-Inflammatory Colonic CD4+ T Lymphocytes in Ulcerative Colitis

**DOI:** 10.1093/ecco-jcc/jjw189

**Published:** 2016-12-07

**Authors:** Christopher A. Lamb, John C. Mansfield, Gaik W. Tew, Deena Gibbons, Anna K. Long, Peter Irving, Lauri Diehl, Jeff Eastham-Anderson, Maria B. Price, Graeme O’Boyle, David E. J. Jones, Sharon O’Byrne, Adrian Hayday, Mary E. Keir, Jackson G. Egen, John A. Kirby

**Affiliations:** a Institute of Cellular Medicine, Newcastle University, Newcastle upon Tyne NE2 4HH, UK; b Department of Gastroenterology, Newcastle upon Tyne Hospitals NHS Foundation Trust, Newcastle upon Tyne NE1 4LP, UK; c Institute of Genetic Medicine, Newcastle University, Newcastle upon Tyne NE1 3BZ, UK; d Research & Early Development, Genentech, South San Francisco, CA 94080, USA; e Peter Gorer Department of Immunobiology, King’s College London, London SE1 9RT, UK; f London Research Institute, Cancer Research UK, London WC2, UK; g Department of Cellular Pathology, Newcastle upon Tyne Hospitals NHS Foundation Trust, Newcastle upon Tyne NE1 4LP, UK; h Department of Gastroenterology, Guys and St Thomas’ NHS Foundation Trust, London SE1 7EH, UK

**Keywords:** ulcerative colitis, inflammatory bowel disease, αEβ7 integrin, CD103, etrolizumab, intraepithelial lymphocytes, Th1, Th17, Th17/1, regulatory T cell, Treg, cytokine, CD4+ T cell, mucosal immunology, effector T cell, helper T cell

## Abstract

**Background and Aims:**

The αEβ7 integrin is crucial for retention of T lymphocytes at mucosal surfaces through its interaction with E-cadherin. Pathogenic or protective functions of these cells during human intestinal inflammation, such as ulcerative colitis [UC], have not previously been defined, with understanding largely derived from animal model data. Defining this phenotype in human samples is important for understanding UC pathogenesis and is of translational importance for therapeutic targeting of αEβ7–E-cadherin interactions.

**Methods:**

αEβ7+ and αEβ7− colonic T cell localization, inflammatory cytokine production and expression of regulatory T cell-associated markers were evaluated in cohorts of control subjects and patients with active UC by immunohistochemistry, flow cytometry and real-time PCR of FACS-purified cell populations.

**Results:**

CD4+αEβ7+ T lymphocytes from both healthy controls and UC patients had lower expression of regulatory T cell-associated genes, including FOXP3, IL-10, CTLA-4 and ICOS in comparison with CD4+αEβ7− T lymphocytes. In UC, CD4+αEβ7+ lymphocytes expressed higher levels of IFNγ and TNFα in comparison with CD4+αEβ7− lymphocytes. Additionally the CD4+αEβ7+ subset was enriched for Th17 cells and the recently described Th17/Th1 subset co-expressing both IL-17A and IFNγ, both of which were found at higher frequencies in UC compared to control.

**Conclusion:**

αEβ7 integrin expression on human colonic CD4+ T cells was associated with increased production of pro-inflammatory Th1, Th17 and Th17/Th1 cytokines, with reduced expression of regulatory T cell-associated markers. These data suggest colonic CD4+αEβ7+ T cells are pro-inflammatory and may play a role in UC pathobiology.

## 1. Introduction

Expression of the β7 integrin on lymphocytes mediates critical functions in mucosal immunity. This subunit is only present in two heterodimeric integrins, α4β7 and αEβ7.^1^ Therapeutic targeting of α4β7:MAdCAM-1-mediated lymphocyte trafficking to the gut in inflammatory bowel disease [IBD] has been clinically validated. Treatment with natalizumab, a humanized monoclonal antibody directed against the α4 integrin subunit, demonstrated clinical efficacy in Crohn’s disease,^2,3^ but was also associated with progressive multifocal leukoencephalopathy,^4^ presumably resulting from impaired α4β1 integrin-mediated lymphocyte homing to the central nervous system and/or mobilization of CD34+ JC virus-containing stem cells from bone marrow.^5^ More recently, vedolizumab, a humanized monoclonal antibody specific for the α4β7 integrin heterodimer, demonstrated efficacy in both Crohn’s disease and UC.^6,7^

In addition to the α4β7 integrin heterodimer, β7 integrin can also pair with αE [CD103]. The resulting αEβ7 integrin heterodimer is predominantly expressed on mucosal lymphocytes and dendritic cells,^8^ where it plays a role in cellular retention through adhesive interactions with E-cadherin expressed on the surface of epithelial cells.^9^ Transcription of the αE integrin subunit is induced by transforming growth factor [TGF]β1,^10^ which is highly expressed in mucosal tissues by epithelial cells,^11^ suggesting that differentiation to and maintenance of an αEβ7+ phenotype occurs in proximity to the epithelial compartment. In support of this, >85% of intraepithelial lymphocytes [IELs] and ~40% of lamina propria lymphocytes [LPLs] in the small intestine are αEβ7+.^12^ αE-deficient mice have reduced numbers of IELs and LPLs in the intestine, while Peyer’s patches [PPs] and splenic lymphocyte populations remain intact.^13^ Additionally, β7-deficient mice lack both α4β7 and αEβ7 integrin expression and have severely impaired gut-tropic lymphocyte migration, leading to poorly developed PPs with diminished cellularity.^14^

Although αEβ7+ cells have been studied in mouse models,^15–22^ there are only limited data on these cells in the human gut, and the role of these cells in human colitis is not understood. Several studies in mice have highlighted αE expression as a marker of distinct and highly suppressive regulatory T cell [TReg] populations, although suppressive activity is also observed in αE− populations.^15–18^ Highlighting the potential for αE to mark effector populations in murine studies, αE blockade can be beneficial in mouse models of colitis,^19^ graft-versus-host disease-associated intestinal inflammation^20^ and solid organ transplant.^21,22^ The role that αEβ7+ T cells play in the normal and inflamed human intestine is still unclear.

Etrolizumab is a humanized monoclonal antibody specific for the β7 integrin subunit and therefore targets both intestinal lymphocyte recruitment and retention by binding, respectively, the α4β7 and αEβ7 integrins. This is in contrast to the mechanism of action of natalizumab and vedolizumab that target lymphocyte recruitment alone by binding α4- and α4β7-mediated homing, respectively. The additional blockade of αEβ7 integrin represents a unique potential mechanism of action, and this therapeutic approach has shown efficacy in moderate to severely active UC.^23,24^ The relative impact of etrolizumab-mediated α4β7 vs αEβ7 integrin blockade on patient response is at present unclear. However, a retrospective analysis of Phase II data in UC observed increased clinical remission among patients with higher expression levels of αE at baseline in comparison to patients with lower levels of αE at baseline, suggesting that αE+ cells may be predictive for response to drug therapy.^24,25^ Understanding the role of αEβ7+ T cells in humans is essential to safely and most effectively use etrolizumab therapy in the correct patients and to the best advantage.

To better understand the role of these cells in intestinal inflammation, we evaluated expression of inflammatory and regulatory markers in αEβ7+ and αEβ7− colonic T lymphocytes in patients with active UC in comparison to healthy control subjects. This analysis of αEβ7+ and αEβ7− colonic T lymphocytes will help to understand the role of the αE integrin in disease pathogenesis, as well as offering insight into the mechanism of etrolizumab and the potential role of this integrin as a biomarker for therapeutic stratification in IBD.

## 2. Methods

### 2.1. Participants and disease stratification

Patients with active UC on stable therapy and healthy control subjects [Table 1] were enrolled in prospective tissue repositories at Newcastle University and King’s College London [KCL], UK. Specimens from the Newcastle cohort were investigated for protein expression by immunohistochemistry and flow cytometry, and specimens from the KCL cohort were investigated for gene expression by quantitative polymerase chain reaction [qPCR]. The aim of this approach was to confirm cellular phenotypic associations by multiple scientific modalities and in distinct geographical patient populations. Enrolled healthy control subjects had normal colonic mucosa undergoing routine endoscopy [e.g. for investigation of iron deficiency anaemia or for polyp surveillance]. All patients were naïve to therapies targeting integrins [etrolizumab, vedolizumab and natalizumab], and tumour necrosis factor [TNF]α [infliximab and adalimumab]. UC patients were considered to have active disease if they had a Mayo endoscopic subscore^26^ of 1 or more; UC patients with endoscopic subscores of 0 were not included in this study. In total, 35 control subjects and 27 UC patients provided tissue for research. These studies were performed according to the principles of the *Declaration of Helsinki*. Written informed consent was obtained in accordance with research and ethics committee [REC] approval [Newcastle and North Tyneside 1 REC 10/H0906/41, Newcastle and North Tyneside 2 REC 22/02 and Wandsworth REC 07/H0803/237].

**Table 1.** Summary of patient demographics.

	Control	UC
Patients [*n*]	35	27
Mayo endoscopy subscore [*n*]:		
0		0
1		6
2		16
3		5
Gender: male/female [*n*]	19/16	17/10
Mean age at biopsy, years [range]	55 [21–77]	45 [19–73]
Mean duration of disease, years [range]		9.8 [0.2–33]
Concomitant IBD medications [*n*]		
Anti-TNF [infliximab, adalimumab]		0
Anti-leukocyte trafficking [etrolizumab, vedolizumab, natalizumab]		0
Immunomodulators [azathioprine, mercaptopurine, methotrexate, MMF]		8 [5, 0, 2, 1]
Systemic corticosteroid [oral prednisolone, intravenous hydrocortisone]		6 [5, 1]
Topical corticosteroid		4
5ASA		20
Topical 5ASA		2
No IBD meds		3

### 2.2. Tissue handling

Colonic mucosal biopsies were obtained at the time of colonoscopy or flexible sigmoidoscopy, and collected from the distal colon to avoid anatomical variations in lymphocyte phenotype or frequency. Between four and eight biopsies were collected per patient with a yield of approximately 75000–100000 T cells from each biopsy. Specimens for immunohistochemistry [IHC] analysis were fixed immediately in neutral buffered formalin prior to paraffin embedding. Fresh tissue was stored in Hank’s balanced salt solution [HBSS] with or without calcium or magnesium [Gibco, Carlsbad, CA, USA] on ice for up to 2 h until enzymatic dissociation.

### 2.3. Colonic biopsy dissociation and lymphocyte enrichment

Biopsies were washed in HBSS containing 5 mM 1,4-dithiothreitol [DTT, Sigma-Aldrich, St Louis, MO, USA] prior to enzymatic tissue dissociation using 10% complete RPMI containing 1.5 mg/ml Collagenase VIII [Sigma-Aldrich] and 50 microg/ml DNase I [Roche, Penzberg, Germany]. The resultant cell suspension was filtered through a 40-μm cell strainer and washed with FACS buffer to collect any residual cells prior to centrifugation and staining.

### 2.4. Cell surface and intracellular protein quantification by flow cytometry

Viable cells were identified using a Live/Dead fixable aqua dead cell stain kit [Invitrogen, Grand Island, NY, USA] according to the manufacturer’s instructions. For identification of cell surface proteins, cells were suspended in 2% fetal calf serum PBS and incubated for 10 min with human FcR blocking reagent [Miltenyi Biotec, Bergisch Gladbach, Germany], prior to the addition of optimized concentrations of fluorochrome-tagged anti-αE, -β7, -α4β7, -CD45, -CD3, -CD4, -CD8a, -CD161 or isotype control antibodies and incubated for 20 min [Supplementary Table 1]. Gating strategies to identify lymphocyte populations are shown in Supplementary Fig 1.

For identification of intracellular proteins, *ex-vivo* cells were left unstimulated or stimulated at 37 °C in 5% CO_2_ for 5 h in the presence of 10 ng/ml phorbol 12-myristate 13-acetate [PMA] and 1 μg/ml ionomycin, with the addition of 10 ng/ml brefeldin A [all Sigma Aldrich] after 1 h. Cells were fixed for 30 min in 4% paraformaldehyde fixation buffer [Biolegend, San Diego, CA, USA], prior to washing with and suspending in permeabilization buffer [eBioscience, San Diego, CA, USA], and then stained for 30 min with optimized concentrations of anti-IL-17A, -IFNγ, -TNFα or isotype control antibodies [Supplementary Table 1].

Data were acquired using a BD FACSCanto II flow cytometer using FACSDiva software v6 [Becton Dickinson, Franklin Lakes, NJ, USA]. Analysis was performed using FlowJo software v9.4.7 [TreeStar, Ashland, OR, USA]. Cell population gates were established based on levels of background fluorescence in isotype control stained, or unstimulated samples.

### 2.5. qPCR analysis of colonic T cell subsets

T lymphocytes isolated from colonic biopsies and identified by flow cytometry as previously described^25^ were sorted using a Becton Dickinson FACS Aria II [SORP] cell sorter into four groups [CD4+αEβ7+, CD4+αEβ7−, CD8+αEβ7+ and CD8+αEβ7−] directly into RLT lysis buffer [Qiagen, Hilden, Germany] and stored at −80 °C prior to RNA extraction. cDNA synthesis was performed using the High-Capacity cDNA Reverse Transcription Kit [Life Technologies, Carlsbad, CA, USA], and quantitative real time PCR analysis was performed using the BioMark HD System [Fluidigm, South San Francisco, CA, USA] using Taqman gene expression of specified genes [Life Technologies]. All gene expression values were normalized to GAPDH expression using the ΔCT method.

### 2.6. Immunohistochemistry staining and quantification

IHC was performed on formalin-fixed and paraffin-embedded colonic biopsies. Blocks were cut into 4-μm sections and stained using a BenchMark Ultra autostainer [Ventana Medical Systems, Tucson, AZ, USA] with optimized concentrations of anti-αE integrin, FOXP3, CD3, CD4 and CD8 antibodies **[** Supplementary Table 2 **]**. Slides were developed with Alkaline Phosphatase Red [Ventana Medical Systems] and Vina Green [Biocare Medical, Concord, CA, USA] chromogen kits.

Whole slide images were acquired with a Nanozoomer 2.0-HT automated slide-scanning platform [Hamamatsu, Hamamatsu City, Japan] at 200× final magnification. Scanned images were analysed in the Matlab software package vR2012b [Mathworks, Natick, MA, USA].

Images from IHC slides were captured on an Olympus BX61 microscope [Olympus, Center Valley, PA, USA] outfitted with a Nuance Multispectral Imaging System [Perkin Elmer, Waltham, MA, USA]. A spectral library was created based on single colour pixel selection from each individual chromogen, allowing unmixing of multiple spatially co-localized chromogens. For each slide, a series of images was collected at a final magnification of 200x with exposure of 160 ms and red, green and haematoxylin were unmixed. Using the spectrally unmixed images as a guide, areas were manually selected on the whole-slide scans specific to either single chromogen or co-localized chromogen. These areas were used to train a Support Vector Machine [SVM] on the RGB colour profile specific to those areas. Application of this SVM to the entire slide image resulted in three binary masks, one for each single or co-localized chromogen. Individual cell borders were segmented as described,^27^ and then scored IHC positive according to the greatest contingent of staining present within its borders, with co-localized area given twice the weight of either single chromogen, as well as at least ~10 µm^2^ of staining present.

Crypt epithelial areas were identified using a combination of an SVM and Genetic Programming [GP]. First, a training set of representative areas was generated manually and assigned a binary classification [positive for crypt lumen, or negative for regions to exclude]. Then, an SVM was trained using RGB^28^ and texture values from these selections. GP was used to determine a sequence of simple morphological operators that maximized the solution accuracy for both positive and negative selections. The resulting SVM and GP algorithm was then applied to the entire set of images. Post-processing, any individual samples with less than 15 000 µm^2^ of total crypt area, or approximately three small crypts, were removed from the analysis. The layer of cell nuclei immediately adjacent to the crypt lumen was identified by dilating the lumen areas approximately one cell diameter, and reconstructing the overlapping cells.

### 2.7. Statistics

Statistical analysis was performed using Prism5 [GraphPad software, San Diego, CA, USA] and JMP11 [SAS, Cary, NC, USA]. The distributions of the samples were assessed for normality using the D’Agostino–Pearson omnibus test. Tukey box plots depict IHC and flow cytometry data. Intra-individual and inter-individual flow cytometry data were compared using paired and unpaired Student’s *t*-tests, respectively. Interleaved scatter plots depict qPCR data, and dotted lines on graphs represent the lower limit of detection for genes of interest. The Mann–Whitney U test was used to compare gene expression data for αE+ vs αE− cell populations. No adjustment for multiple comparisons was performed, except where noted in the text. Statistical significance [*p* < 0.05] was defined based on nominal *p*-values, and different levels were noted on graphs as follows: *p < 0.05, **p < 0.01, ***p < 0.001, ****p < 0.0001.

## 3. Results

### 3.1. UC is associated with an expansion of αE− FOXP3+ cells in the lamina propria

Human studies have previously demonstrated an influx of TReg to the intestinal mucosa in active IBD by a combination of flow cytometry, IHC and qPCR.^29^ We first sought to determine if T cells were the predominant leukocyte subset expressing αE during active UC, and whether there was any association of αE integrin with markers of regulatory T cell function.

**Figure 1.**
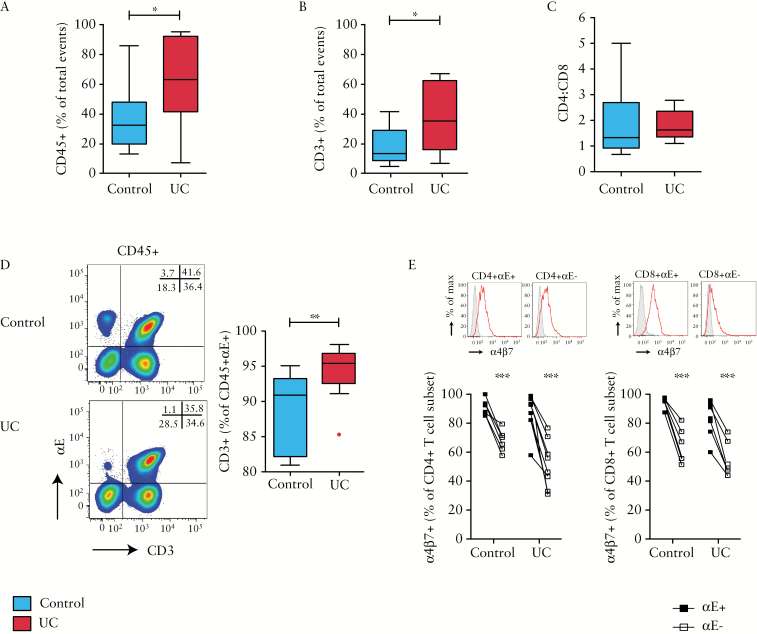
Ulcerative colitis is associated with an influx of mucosal leukocytes. Cells isolated from colonic biopsies sampled from control subjects [*n* = 14] and UC patients [*n* = 10] were evaluated by flow cytometry. Frequency of [A] CD45+ leukocytes, and [B] CD3+ T lymphocytes in the colonic mucosa is shown, along with [C] CD4:CD8 ratio of all CD3+ T cells and [D] representative αE vs CD3 FACS plots of CD45+ leukocytes, with summary graph of the proportion of CD45+αE+ leukocytes that were T cells. [E] In a subset of patients, surface expression of α4β7 dimer on αE+ and αE− T cells from control subjects [*n* = 6] and UC patients [*n* = 8] was examined.

Flow cytometry demonstrated a significant increase in total CD45+ leukocytes and CD3+ T cells in active UC in comparison to healthy subjects [Figure 1A&B] without a difference in CD4:CD8 ratio [Figure 1C]. Of all αE+ leukocytes, CD3+ T cells represented the dominant leukocyte subset [Figure 1D]. In control subjects nearly 90% of αE+ leukocytes were T cells, significantly rising to nearly 95% in UC. The frequency of α4β7 integrin expression was higher on αE+ relative to αE− T cells in both UC patients and controls [Figure 1E]. In addition, not all αE+ T cells expressed α4β7 as up to 40% of αE+ T cells in patients with active UC did not express α4β7 [Figure 1E].

**Figure 2.**
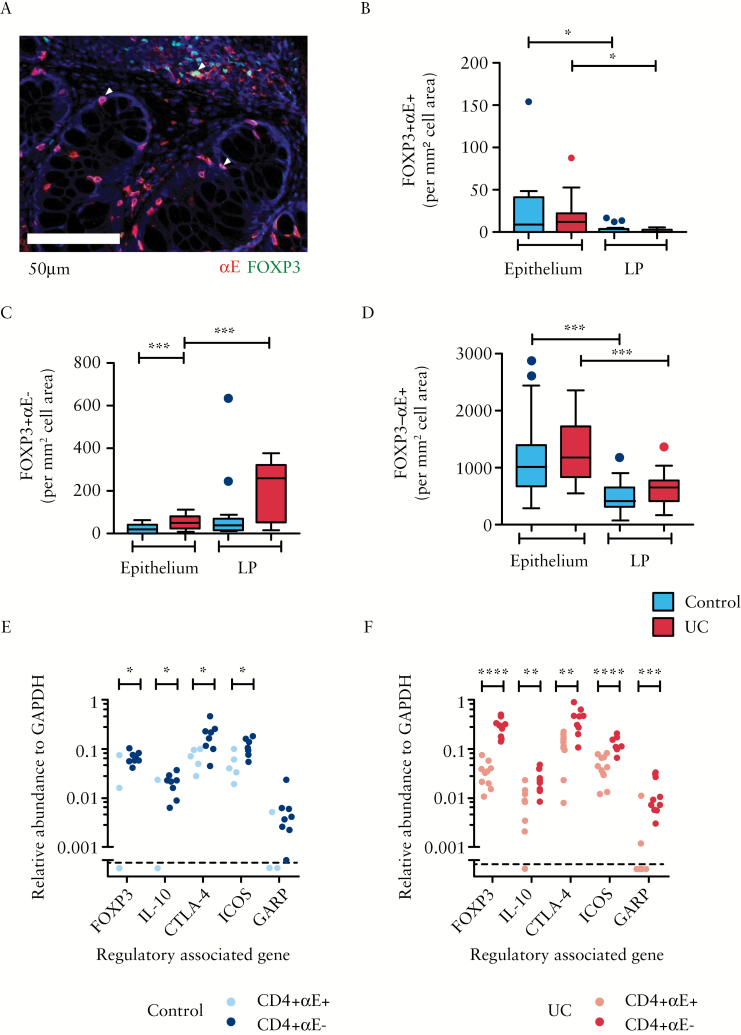
Lymphocytes with a regulatory phenotype are expanded in the colonic lamina propria [LP] during ulcerative colitis and lack αE integrin. Dual stain IHC was performed on formalin-fixed paraffin-embedded colonic biopsies to evaluate co-expression of the αE integrin [red] with FOXP3 [green]. Single and dual expressing cells [yellow, indicated by arrowheads] were enumerated using the Nuance Multispectral Tissue Imaging System combined with automated morphometric analysis. [A] A representative Nuance spectrally unmixed image is shown for a patient with active UC. Scale bar represents 50 μm. Automatic cell counting was used to enumerate [B] FOXP3+αE+, [C] FOXP3+αE− cells and [D] FOXP3−αE+ cells per mm^2^ of cell area within the epithelium or LP in control subjects [*n* = 16] and patients with active UC [*n* = 14]. In a separate study cohort, FACS-sorted unstimulated CD4+αE+ and CD4+αE− colonic lymphocytes were analysed for TReg gene expression in [E] control subjects [*n* = 8] and [F] patients with active UC [*n* = 10].

We next examined the expression of αE integrin and the regulatory transcription factor FOXP3 using dual colour IHC. Consistent with preferential localization of αE+ cells to anatomical sites rich in E-cadherin, αE was predominantly observed on epithelial-associated CD3+, CD4+ and CD8+ cells with a high proportion of cells showing co-staining for T cell markers and αE [Supplementary Figure 2A–C]. Very few αE+FOXP3+ cells were observed in either the epithelium or the LP, with a small but significant increase in epithelial-associated cells [Figure 2A&B]. In contrast to the relative scarcity of αE+FOXP3+ cells, there was a marked expansion of FOXP3+ cells lacking the αE integrin in the LP of patients with active UC [Figure 2C]. The majority of αE+ cells were observed to be FOXP3−, with preferential localization to the epithelium [Figure 2D].

### 3.2. Human colonic CD4+αE− T cells display enhanced levels of TReg-associated genes compared with CD4+αE+ cells

An expansion of FOXP3+ cells in the LP during inflammation may be indicative of infiltrating/expanding TReg populations. However, activated effector T cells can express FOXP3^30^ and so the transcription factor is not specific enough by itself to identify TReg. Human TReg have been shown to have high expression of IL-10, CTLA4, ICOS and GARP in addition to FOXP3.^31–33^ Therefore, gene expression of these TReg-associated markers was evaluated in purified αE+ and αE− subsets of both CD4+ and CD8+ TCRα β+ T cells. Consistent with the IHC analysis, CD4+αE− T lymphocytes consistently expressed higher levels of TReg-associated genes in comparison to CD4+αE+ cells. Both in control subjects and more strikingly in UC patients, sorted CD4+αE− T cells had significantly higher levels of FOXP3, IL-10, CTLA4 and ICOS in comparison with CD4+αE+ T cells [Fig 2E&F]. When correction was made for multiple comparisons using the Benjamini–Hochberg false detection rate adjustment of 10%, FOXP3 remained significantly elevated in CD4αE– vs CD4+αE+ T cells in both UC and control subjects. No difference in expression of TReg-associated genes was noted between CD8+αE+ and CD8+αE– lymphocytes in either group [Supplementary Figure 3A].

### 3.3. Expression of the αE integrin by colonic CD4+ T cells is associated with Th17 differentiation

These findings suggest that, in contrast to the mouse, αE is not a marker of TReg in the human colon. We next investigated if αE was associated with a pro-inflammatory T cell phenotype.

**Figure 3.**
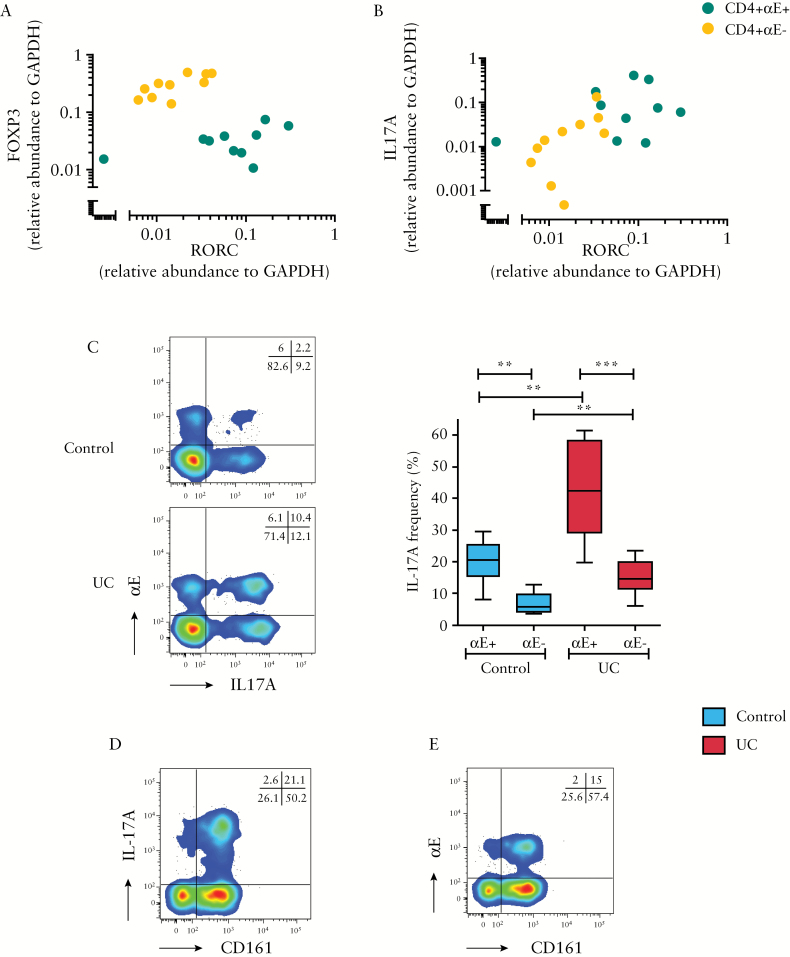
αE integrin expression by colonic CD4+ T lymphocytes is associated with Th17 differentiation. FACS-sorted, unstimulated CD4+αE+ and CD4+αE− T lymphocytes isolated from active UC colonic biopsies were analysed for gene expression of [A] FOXP3 vs RORC and [B] IL17A vs RORC [*n* = 10 patients]. [C] In a separate study cohort, IL-17A protein expression in CD4+αE+ and CD4+αE− T cells was evaluated by FACS in colonic *ex-vivo* stimulated T lymphocytes from control subjects [*n* = 6] and patients with active UC [*n* = 8]. Representative two-colour FACS plots from control and UC patients are shown alongside summary Tukey box plots. Representative flow cytometry showing CD4+ T cell CD161 co-expression with [D] intracellular IL-17A, and [E] surface αE staining on a patient with active UC.

The RORC gene encodes a member of the nuclear hormone receptor family of transcription factors, and is an important component of Th17 differentiation which has been associated with IBD in a number of studies.^34,35^ Relative gene expression levels of FOXP3, RORC and IL17A in CD4+αE+ and CD4+αE– T cells were therefore examined in our active UC cohort. In contrast to CD4+αE+ lymphocytes, CD4+αE– T lymphocytes had lower levels of RORC and IL17A, along with higher levels of FOXP3 expression [Fig 3A&B], suggesting that these CD4 subsets consist of T cells in unique states of differentiation.

To confirm the association of Th17 phenotype with αE integrin expression, cellular cytokine production was examined by flow cytometry. Analysis of CD4+αE+ colonic lymphocytes revealed a 2- to 3-fold increased frequency of IL-17A+ cells in comparison with CD4+αE− lymphocytes [Figure 3C]. Furthermore, the frequency of IL-17A was twice as high in active UC CD4+αE+ cells compared to control subject CD4+αE+ lymphocytes. Consistent with previous research showing that IL-17-producing T cells are enriched in the CD161+ population, the majority of CD4 cells expressing either IL-17A or αE also expressed surface CD161 [Figure 3D&E].^36^

In the CD8 compartment, αE+ cells from patients with active UC demonstrated a higher gene expression of IL-17A compared with CD8+αE− T cells, while no difference in gene expression was noted in control subjects [Supplementary Figure 3B]. However, FACS analysis of colonic *ex vivo* stimulated CD8+ T cells demonstrated very low frequency of IL-17A cytokine-producing cells irrespective of disease status or αE expression [Supplementary Figure 4A].

### 3.4. Higher production of Th1 cytokines is observed in CD4+αE+ relative to CD4+αE− colonic T cells

**Figure 4.**
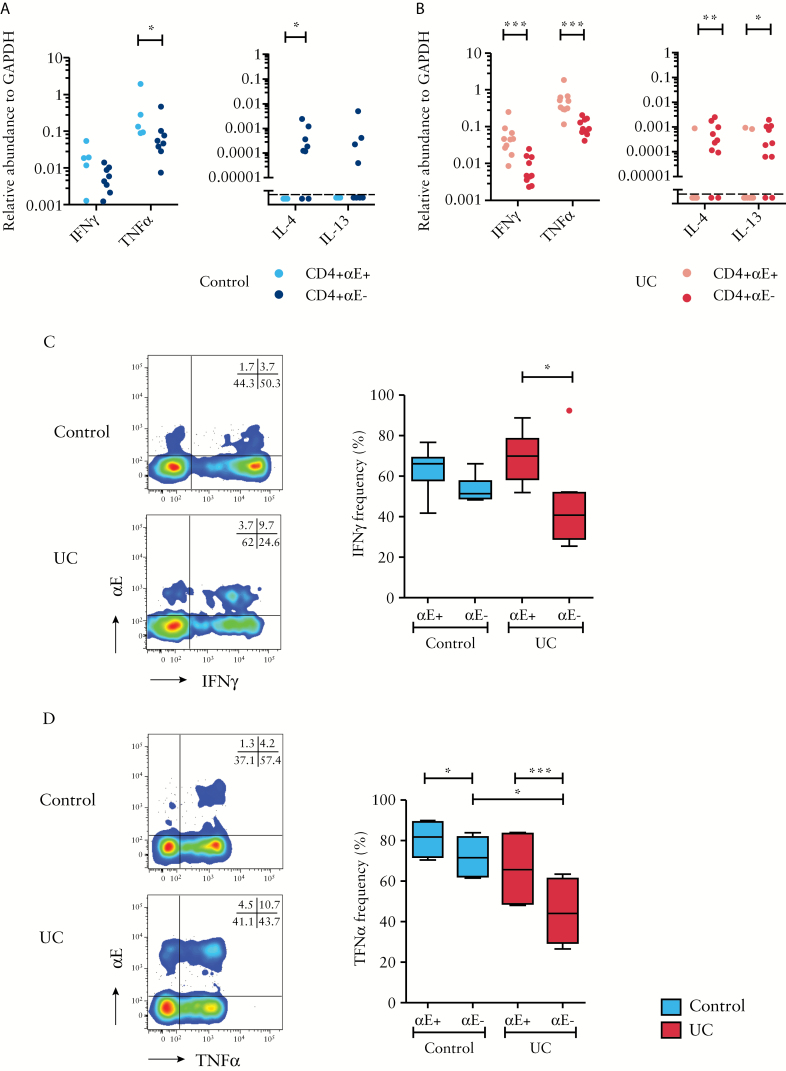
Colonic CD4+αE+ lymphocytes express higher levels of Th1 cytokines relative to CD4+αE− lymphocytes. FACS-sorted CD4+αE+ and CD4+αE− T lymphocytes from colonic biopsies harvested from [A] control subjects [*n* = 8] and [B] patients with active UC [*n* = 10] were evaluated for gene expression of IFNγ, TNFα, IL-4 and IL-13. In a separate study cohort, *ex-vivo* stimulated T cells were evaluated by flow cytometry for intracellular [C] IFNγ [*n* = 6 control subjects and *n* = 8 patients with active UC], and [D] TNFα protein expression [*n* = 4 control subjects and *n* = 6 patients with active UC]. Representative two-colour FACS plots from control and UC patients are shown alongside summary Tukey box plots.

Given the association between Th17 differentiation and αE integrin, we next sought to determine whether CD4+αE+ T cells also had a Th1 phenotype using qPCR examination of T cells and flow cytometry of PMA/ionomycin-stimulated colonic T cells. Sort-purified CD4+αE+ T cells from control subjects demonstrated higher gene expression of TNFα compared with CD4+αE− T cells. A trend to higher IFNγ expression by CD4+αE+ T cells in control subjects was also observed [*p* = 0.09, Figure 4A].

The relationship of higher effector gene expression of sort-purified CD4+αE+ T cells in comparison to CD4+αE− T cells was more striking in active UC patients [Figure 4B]. The frequency of IFNγ protein-expressing cells and the level of IFNγ transcripts were significantly increased in CD4+αE+ compared to CD4+αE− cells in UC patients, and the frequency of IFNγ-producing cells was overall decreased in the CD4+αE− population in patients with UC [Figure 4C]. CD8+αE+ T cells also had increased IFNγ-expressing potential compared to CD8+αE– cells by protein and gene expression [Supplementary Figures 3B & 4B].

Similar relationships of protein and transcript level increases were observed for TNFα expression in the CD4+αE+ compared to CD4+αE- population [Figure 4A, B&D]. In contrast to these findings in CD4+ T cells, no difference in TNFα protein or gene expression was seen between CD8+αE+ and CD8+αE− cells [Supplementary Figures 3B & 4C].

Although gene expression of the Th2 cytokines IL-4 and IL-13 by CD4+ T cells in the colon was very low in both controls and UC, a small but significant increase in IL-4 gene expression in CD4+αE− cells relative to CD4+αE+ cells was observed [Figure 4A&B] in control subjects and UC patients. An increase in IL-13 gene expression was also seen in CD4+αE- cells relative to CD4+αE+ cells in active UC.

### 3.5. CD4+αE+ T cells display a Th17/Th1 phenotype that is exaggerated in UC

**Figure 5.**
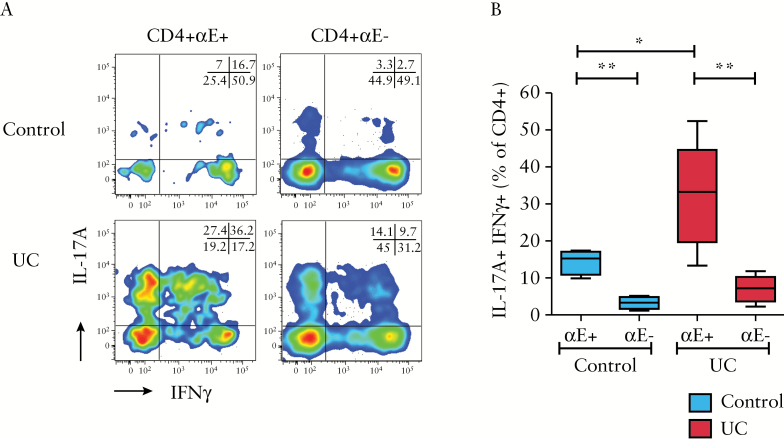
αE integrin expression by CD4+ T lymphocytes is associated with a Th17/Th1 phenotype, which is enhanced in ulcerative colitis. *Ex-vivo* colonic CD4+ T cells were stimulated and analysed by flow cytometry for dual cytokine production of IL-17A and IFNγ. [A] Representative two-colour FACS plots of IL-17A vs IFNγ for CD4+αE+, CD4+αE− cells. [B] Summary Tukey box plots of double positive IL-17A+IFNγ+ lymphocyte frequency and double negative IL-17A−IFNγ− lymphocyte frequency [*n* = 4 control subjects and *n* = 6 UC patients].

Recent studies have identified that in contrast to Th1 cells, which demonstrate stable lineage commitment after differentiation from naïve cells, Th17 cells retain some degree of plasticity which can give rise to so-called Th17/Th1 dual IL-17A-positive and IFNγ-positive cells.^37^ These potentially pathogenic cells have previously been identified in Crohn’s disease.^38^ We therefore used flow cytometry to explore if Th17/Th1 cells were present in UC and if there was any differential phenotype relative to αE expression. Dual IL-17A+IFNγ+ T cells were identified in control subjects and active UC [Figure 5A&B]. Notably, the frequency of dual cytokine-producing CD4+αE+ cells was increased 2-fold in UC compared to control tissue samples. CD8 lymphocytes had a very low potential to concurrently produce both IL-17A and IFNγ [Supplementary Figure 4D].

## 4. Discussion

A substantial fraction of gut-associated T lymphocytes express the αE integrin, yet the phenotype of these cells in humans and their potential contribution to diseases such as UC remain largely unknown. TGFβ, which plays a key role in the differentiation of both Th17 cells and induced TRegs depending on the cytokine milieu,^37,39^ also induces αE expression on T cells. In this study, utilizing two independent cohorts, one examined for protein expression by IHC and FACS and one examined for gene expression by qPCR, we consistently observed CD4+αE+ lymphocytes to be enriched in mucosal effector T cell populations expressing several inflammatory cytokines, including Th17 and Th1, but not Th2. The recently described Th17/Th1 lineage cells, which express T-bet and RORC and are potentially potent effector T cells, were also increased within CD4+αE+ cells.^37^ Consistent with their increased expression of IL-17A, we found that the majority of CD4+αE+ T cells also express CD161, a surface marker previously found to be highly enriched in the Th17 subset.^36^ These data suggest that TGFβ-driven differentiation into the Th17 lineage, rather than the Treg lineage, is favoured in CD4+αE+ T cells in the gut. Previous studies have identified Th17/Th1 lineage cells that are enriched in Crohn’s disease patients.^37^ Our data extend these findings into UC, indicating that Th17/Th1 cells are present in UC and are most frequent in the CD4+αE+ population. In addition, CD4+αE+ cells in UC had higher transcription and expression of the pro-inflammatory cytokine TNFα, which although classically associated with Th1 cells, can also be produced by Th17 cells,^40^ suggesting that the pro-inflammatory functions of these cells may extend beyond production of only IL-17A and IFNγ.

As we observe substantial enrichment of regulatory markers in αE− human intestinal T cells, αE integrin is probably not an exclusive marker for regulatory T cells in humans. TRegs can suppress effector functions of other T lymphocytes, in part through the production of IL-10 and TGFβ1. In mice, TReg populations expressing αE have been identified and shown to suppress inflammation in a number of disease models, including colitis,^17^ chronic graft versus host disease^18^ and antigen-induced arthritis.^15^ In human UC and control subjects we found CD4+αE+ lymphocytes have reduced transcription of the regulatory T cell-associated gene FOXP3 relative to their CD4+αE− counterparts. Competitive antagonism has previously been demonstrated in transcription of FOXP3 and RORC, stabilizing, respectively, the TReg and Th17 phenotype.^41^ Consistent with these findings, we demonstrated that CD4+ T cells sorted on the basis of αE surface expression and analysed for RORC and FOXP3 gene expression, cluster into RORC^hi^ FOXP3^lo^ [CD4+αE+] and RORC^lo^ FOXP3^hi^ [CD4+αE−] subsets, suggestive of distinct functions. Although FOXP3 may be expressed by activated^30^ or a minor non-regulatory T cell subset,^42^ other regulatory T cell-associated genes [CTLA-4, GARP, ICOS, IL-10] were also upregulated in CD4+αE− T cells. While none of these genes are specific to TRegs, for instance IL-10 can also be produced by activated Th1 cells,^43^ the aggregate pattern of gene expression observed in this study suggests strongly that the majority of human CD4+αE+ T cells do not have an enhanced regulatory function, and that the majority of T cell-mediated immunoregulatory activity is contained within the CD4+αE− population.

Taken together, these data support a model in which the mucosal CD4+αE+ population, already skewed towards an effector T cell phenotype in the steady state, becomes highly enriched in cells capable of producing pro-inflammatory cytokines. These cytokines have been shown to contribute to the epithelial damage and dysfunction characteristic of UC.^44–46^ The mechanisms responsible for promoting the differentiation and activation of CD4+αE+ T cells *in vivo* remain largely unknown, but could involve factors directly produced by intestinal epithelial cells such as TGFβ1, which can induce αE expression and a Th17 phenotype.^10,47^ Thus, cross-talk between CD4+ mucosal lymphocytes and dysfunctional epithelial cells may contribute to the pathobiology of UC.^9^

Etrolizumab has recently demonstrated efficacy during a Phase II clinical trial in moderate to severe UC.^24^ This monoclonal antibody is selective for the β7 integrin subunit and thus targets both αEβ7 and α4β7 integrin-expressing cells. Notably, etrolizumab treatment was associated with a significant reduction in the number of αE+ cells within the colonic epithelial compartment of patients responding to therapy. In addition, post-hoc analysis demonstrated higher remission rates in patients with higher baseline colonic αE expression as measured by IHC or whole-biopsy qPCR.^24,25^ Together with data presented in the current study, these findings suggest that blockade of αEβ7:E-cadherin interactions by etrolizumab may interfere with epithelial cell–lymphocyte localization^48^, and could function in combination with inhibition of α4β7:MAdCAM-mediated lymphocyte trafficking, or independently of α4β7 where αEβ7 is expressed in isolation, to reduce colonic inflammation in UC.^24^ It is not clear whether αE:E-cadherin interactions are required for effector T cell function, or whether αE is simply a biomarker of a cytokine-responsive mucosa-associated cell. Specifically, our study identified very limited phenotypic differences between αE+ and αE− colonic CD8+ T cells. However, αE is likely to influence the spatial localization of CD8+ T cells relative to the epithelium and the immunological synapse may influence release of cytotoxic granules. Thus, further study will be required in larger patient groups as well as additional studies focused on identifying the pathways responsible for CD4+αE+ and CD8+αE+ T cell recruitment, differentiation and activation in the absence and presence of factors blocking interaction of the integrin with E-cadherin.

The goal of personalized medicine in heterogeneous diseases such as UC, in which treatment paradigms are based on knowledge of the underlying biology of disease using testable biomarkers or genetics, will require careful and iterative investigation of pathobiology in the context of clinical trials. Our findings highlight the importance of establishing the function of cell populations in directly relevant human tissues and patient groups. Mixed results have been reported for the efficacy of targeting β7 integrins or corresponding cell adhesion molecules in murine models of IBD,^49–54^ and one possible explanation is key species differences in function of leukocyte populations that are blocked from entry or retention within the gut. Differences in human cell biology demonstrated in this study from previous reported murine research highlight the importance of directly evaluating target cell populations in patient samples to ultimately enable appropriate patient selection strategies. The specific contribution of αE+ T cells to maintenance and loss of intestinal homeostasis will need to be evaluated in the context of integrin blockade in patients, ideally in ongoing Phase III studies, to further clarify the mechanisms by which integrin-targeted therapies function in UC, and whether these cells may be of interest for patient-selection strategies.

## Funding

This work was supported by the Wellcome Trust [grant number 093885/Z/10/Z to CAL]; by the Biomedical Research Centre of Guy’s and St Thomas’ Hospitals and King’s College London, by the NIHR Newcastle Biomedical Research Centre, and by Genentech Inc. CAL is a Clinical Lecturer supported by the National Institute for Health Research [NIHR].

## Conflict of Interest

During this study GWT, JEA, LD, SO’B, MEK and JGE were full-time employees of Genentech, Inc., a member of the Roche Group. Financial or non-financial research support, educational funds or compensation for consulting, speaking or teaching has been received from: CAL: Genentech, Techlab, Immundiagnostik, Roche Tissue diagnostics, Takeda; JCM: AbbVie, Ferring, Genentech, Takeda; DG: Genentech, Cerimon Pharmaceuticals; PI: Abbvie, MSD, Ferring, Vifor, Genentech, Takeda, Warner Chilcott, Shire, Johnson & Johnson, Falk, Pharmacosmos; GO’B: Genentech; DEJ: Genentech; AH: HS-Lifesciences, ImmunoQure, Genentech, MedImmune; JAK: Genentech, GlaxoSmithKline, Intercept Pharmaceuticals. AL and MBP have nothing to disclose.

## Author Contributions

CAL, JCM, DG, SOB, AH, MEK, JGE and JAK participated in study design. CAL, JCM, AKL, PI and MBP participated in patient recruitment and tissue acquisition. All authors were responsible for data analysis and interpretation. CAL, MEK, JGE and JAK drafted the initial manuscript. All authors participated in subsequent manuscript redrafting. The authors confirm this manuscript has not been previously published and is not under consideration for publication elsewhere.

## Supplementary Data

Supplementary date are available at *ECCO-JCC* online.

## Supplementary Material

Supplementary_data

## References

[1] Gorfu G Rivera-Nieves J Ley K Role of beta7 integrins in intestinal lymphocyte homing and retention. Curr Mol Med 2009;9:836–50. 19860663 10.2174/156652409789105525 PMC2770881

[2] Targan SR Feagan BG Fedorak RN Natalizumab for the treatment of active Crohn’s disease: Results of the encore trial. Gastroenterology 2007;132:1672–83. 17484865 10.1053/j.gastro.2007.03.024

[3] Sandborn WJ Colombel JF Enns R Natalizumab induction and maintenance therapy for Crohn’s disease. N Engl J Med 2005;353:1912–25. 16267322 10.1056/NEJMoa043335

[4] Van Assche G Van Ranst M Sciot R Progressive multifocal leukoencephalopathy after natalizumab therapy for Crohn’s disease. N Engl J Med 2005;353:362–8. 15947080 10.1056/NEJMoa051586

[5] Major EO Progressive multifocal leukoencephalopathy in patients on immunomodulatory therapies. Annu Rev Med 2010;61:35–47. 19719397 10.1146/annurev.med.080708.082655

[6] Feagan BG Rutgeerts P Sands BE Vedolizumab as induction and maintenance therapy for ulcerative colitis. N Engl J Med 2013;369:699–710. 23964932 10.1056/NEJMoa1215734

[7] Sandborn WJ Feagan BG Rutgeerts P Vedolizumab as induction and maintenance therapy for Crohn’s disease. N Engl J Med 2013;369:711–21. 23964933 10.1056/NEJMoa1215739

[8] Kilshaw PJ Alpha e beta 7. Mol Pathol 1999;52:203–7. 10694940 10.1136/mp.52.4.203 PMC395700

[9] Swamy M Jamora C Havran W Hayday A Epithelial decision makers: In search of the ‘epimmunome’. Nat Immunol 2010;11:656–65. 20644571 10.1038/ni.1905 PMC2950874

[10] Al-Hamidi A Pekalski M Robertson H Ali S Kirby JA Renal allograft rejection: The contribution of chemokines to the adhesion and retention of αE(CD103)β7 integrin-expressing intratubular T cells. Mol Immunol 2008;45:4000–7. 18649941 10.1016/j.molimm.2008.06.011

[11] Babyatsky MW Rossiter G Podolsky DK Expression of transforming growth factors alpha and beta in colonic mucosa in inflammatory bowel disease. Gastroenterology 1996;110:975–84. 8613031 10.1053/gast.1996.v110.pm8613031

[12] Cerf-Bensussan N Jarry A Brousse N A monoclonal antibody (HML-1) defining a novel membrane molecule present on human intestinal lymphocytes. Eur J Immunol 1987;17:1279–85. 3498635 10.1002/eji.1830170910

[13] Schon MP Arya A Murphy EA Mucosal T lymphocyte numbers are selectively reduced in integrin α_E_(CD103)-deficient mice. J Immunol 1999;162:6641–9. 10352281

[14] Butcher EC Williams M Youngman K Rott L Briskin M Lymphocyte trafficking and regional immunity. Adv Immunol 1999;72:209–53. 10361577 10.1016/s0065-2776(08)60022-x

[15] Huehn J Siegmund K Lehmann JC Developmental stage, phenotype, and migration distinguish naive- and effector/memory-like CD4+ regulatory t cells. J Exp Med 2004;199:303–13. 14757740 10.1084/jem.20031562 PMC2211798

[16] Banz A Peixoto A Pontoux C A unique subpopulation of CD4+ regulatory T cells controls wasting disease, IL-10 secretion and T cell homeostasis. Eur J Immunol 2003;33:2419–28. 12938218 10.1002/eji.200324205

[17] Lehmann J Huehn J de la Rosa M Expression of the integrin αEβ7 identifies unique subsets of CD25+ as well as CD25- regulatory t cells. Proc Natl Acad Sci U S A 2002;99:13031–6. 12242333 10.1073/pnas.192162899 PMC130581

[18] Zhao D Zhang C Yi T In vivo-activated CD103+CD4+ regulatory T cells ameliorate ongoing chronic graft-versus-host disease. Blood 2008;112:2129–38. 18550852 10.1182/blood-2008-02-140277 PMC2518910

[19] Ludviksson BR Strober W Nishikomori R Hasan SK Ehrhardt RO Administration of mab against αEβ7 prevents and ameliorates immunization-induced colitis in IL-2^-/-^ mice. J Immunol 1999;162:4975–82. 10202045

[20] El-Asady R Yuan R Liu K TGF-β-dependent CD103 expression by CD8^+^ T cells promotes selective destruction of the host intestinal epithelium during graft-versus-host disease. J Exp Med 2005;201:1647–57. 15897278 10.1084/jem.20041044 PMC2212926

[21] Hadley GA Bartlett ST Via CS Rostapshova EA Moainie S The epithelial cell-specific integrin, CD103 (Eintegrin), defines a novel subset of alloreactive CD8+ CTl. J Immunol 1997;159:3748–56. 9378961

[22] Yuan R El-Asady R Liu K Critical role for CD103+CD8+ effectors in promoting tubular injury following allogeneic renal transplantation. J Immunol 2005;175:2868–79. 16116173 10.4049/jimmunol.175.5.2868

[23] Rutgeerts PJ Fedorak RN Hommes DW A randomised phase I study of etrolizumab (rhuMAb β7) in moderate to severe ulcerative colitis. Gut 2013;62:1122–30. 22717454 10.1136/gutjnl-2011-301769 PMC3711369

[24] Vermeire S O’Byrne S Keir M Etrolizumab as induction therapy for ulcerative colitis: A randomised, controlled, phase 2 trial. Lancet 2014;384:309–18. 24814090 10.1016/S0140-6736(14)60661-9

[25] Tew GW Hackney JA Gibbons D Association between response to etrolizumab and expression of integrin alphae and granzyme a in colon biopsies of patients with ulcerative colitis. Gastroenterology 2016;150:477–87 e9. 26522261 10.1053/j.gastro.2015.10.041

[26] Schroeder KW Tremaine WJ Ilstrup DM Coated oral 5-aminosalicylic acid therapy for mildly to moderately active ulcerative colitis. A randomized study. N Engl J Med 1987;317:1625–9. 3317057 10.1056/NEJM198712243172603

[27] Veta M van Diest PJ Kornegoor R Automatic nuclei segmentation in H&E stained breast cancer histopathology images. PLoS One 2013;8:e70221. 23922958 10.1371/journal.pone.0070221 PMC3726421

[28] Wang X-Y Wang T Bu J Color image segmentation using pixel wise support vector machine classification. Pattern Recognit 2011;44:777–87.

[29] Maul J Loddenkemper C Mundt P Peripheral and intestinal regulatory CD4+ CD25(high) T cells in inflammatory bowel disease. Gastroenterology 2005;128:1868–78. 15940622 10.1053/j.gastro.2005.03.043

[30] Wang J Ioan-Facsinay A van der Voort EI Huizinga TW Toes RE Transient expression of FOXP3 in human activated nonregulatory CD4+ T cells. Eur J Immunol 2007;37:129–38. 17154262 10.1002/eji.200636435

[31] Miyara M Yoshioka Y Kitoh A Functional delineation and differentiation dynamics of human CD4+ T cells expressing the FOXP3 transcription factor. Immunity 2009;30:899–911. 19464196 10.1016/j.immuni.2009.03.019

[32] Tran DQ Andersson J Wang R GARP (LRRC32) is essential for the surface expression of latent TGF-beta on platelets and activated FOXP3+ regulatory t cells. Proc Natl Acad Sci U S A 2009;106:13445–50. 19651619 10.1073/pnas.0901944106 PMC2726354

[33] Kornete M Sgouroudis E Piccirillo CA ICOS-dependent homeostasis and function of FOXP3+ regulatory T cells in islets of nonobese diabetic mice. J Immunol 2012;188:1064–74. 22227569 10.4049/jimmunol.1101303

[34] Fujino S Andoh A Bamba S Increased expression of interleukin 17 in inflammatory bowel disease. Gut 2003;52:65–70. 12477762 10.1136/gut.52.1.65 PMC1773503

[35] Seiderer J Elben I Diegelmann J Role of the novel Th17 cytokine IL-17F in inflammatory bowel disease (IBD): Upregulated colonic IL-17F expression in active Crohn’s disease and analysis of the IL17F p.His161Arg polymorphism in IBD. Inflamm Bowel Dis 2008;14:437–45. 18088064 10.1002/ibd.20339

[36] Cosmi L De Palma R Santarlasci V Human interleukin 17-producing cells originate from a CD161+CD4+ T cell precursor. J Exp Med 2008;205:1903–16. 18663128 10.1084/jem.20080397 PMC2525581

[37] Weaver CT Elson CO Fouser LA Kolls JK The Th17 pathway and inflammatory diseases of the intestines, lungs, and skin. Annu Rev Pathol 2013;8:477–512. 23157335 10.1146/annurev-pathol-011110-130318 PMC3965671

[38] Annunziato F Cosmi L Santarlasci V Phenotypic and functional features of human Th17 cells. J Exp Med 2007;204:1849–61. 17635957 10.1084/jem.20070663 PMC2118657

[39] Basu R Hatton RD Weaver CT The Th17 family: Flexibility follows function. Immunol Rev 2013;252:89–103. 23405897 10.1111/imr.12035 PMC3607325

[40] Pelletier M Maggi L Micheletti A Evidence for a cross-talk between human neutrophils and Th17 cells. Blood 2010;115:335–43. 19890092 10.1182/blood-2009-04-216085

[41] Weaver CT Hatton RD Interplay between the Th17 and Treg cell lineages: A (co-)evolutionary perspective. Nat Rev Immunol 2009;9: 883–9. 19935807 10.1038/nri2660

[42] Miyao T Floess S Setoguchi R Plasticity of FOXP3(+) T cells reflects promiscuous FOXP3 expression in conventional T cells but not reprogramming of regulatory tT cells. Immunity 2012;36:262–75. 22326580 10.1016/j.immuni.2011.12.012

[43] Cardone J Le Friec G Vantourout P Complement regulator CD46 temporally regulates cytokine production by conventional and unconventional T cells. Nat Immunol 2010;11:862–71. 20694009 10.1038/ni.1917 PMC4011020

[44] Neurath MF Cytokines in inflammatory bowel disease. Nat Rev Immunol 2014;14:329–42. 24751956 10.1038/nri3661

[45] Goretsky T Dirisina R Sinh P P53 mediates TNF-induced epithelial cell apoptosis in IBD. Am J Pathol 2012;181:1306–15. 22863952 10.1016/j.ajpath.2012.06.016 PMC3463630

[46] Seno H Miyoshi H Brown SL Efficient colonic mucosal wound repair requires TREM2 signaling. Proc Natl Acad Sci U S A 2009;106:256–61. 19109436 10.1073/pnas.0803343106 PMC2629230

[47] Mangan PR Harrington LE O’Quinn DB Transforming growth factor-β induces development of the T_H_17 lineage. Nature 2006;441:231–4. 16648837 10.1038/nature04754

[48] Zundler S Schillinger D Fischer A Blockade of αEβ7 integrin suppresses accumulation of CD8+ and Th9 lymphocytes from patients with IBD in the inflamed gut in vivo. Gut 2016 (in press). 10.1136/gutjnl-2016-312439 27543429

[49] Soriano A Salas A Sans M Vcam-1, but not Icam-1 or Madcam-1, immunoblockade ameliorates DSS-induced colitis in mice. Lab Invest 2000;80:1541–51. 11045571 10.1038/labinvest.3780164

[50] Annacker O Coombes JL Malmstrom V Essential role for CD103 in the T cell-mediated regulation of experimental colitis. J Exp Med 2005;202:1051–61. 16216886 10.1084/jem.20040662 PMC2213206

[51] Apostolaki M Manoloukos M Roulis M Role of β7 integrin and the chemokine/chemokine receptor pair CCL25/CCR9 in modeled TNF-dependent Crohn’s disease. Gastroenterology 2008;134: 2025–35. 18439426 10.1053/j.gastro.2008.02.085

[52] Farkas S Hornung M Sattler C Blocking Madcam-1 in vivo reduces leukocyte extravasation and reverses chronic inflammation in experimental colitis. Int J Colorectal Dis 2006;21:71–8. 15856265 10.1007/s00384-004-0709-y

[53] Waldman E Lu SX Hubbard VM Absence of β7 integrin results in less graft-versus-host disease because of decreased homing of alloreactive T cells to intestine. Blood 2006;107:1703–11. 16291587 10.1182/blood-2005-08-3445 PMC1895413

[54] Liu K Anthony BA Yearsly MM CD103 deficiency prevents graft-versus-host disease but spares graft-versus-tumor effects mediated by alloreactive CD8 T cells. PLoS One 2011;6:e21968. 21779359 10.1371/journal.pone.0021968 PMC3136479

