# Supplementary material for: αEβ7 Integrin Identifies Subsets of Pro-Inflammatory Colonic CD4+ T Lymphocytes in Ulcerative Colitis
Source: J Crohns Colitis. 2016 Dec 7;11(5):610–20. doi: 10.1093/ecco-jcc/jjw189 (PMC5815571; doi:10.1093/ecco-jcc/jjw189)
Supplement: Supplementary_data [file jjw189_suppl_supplementary_data.pdf]

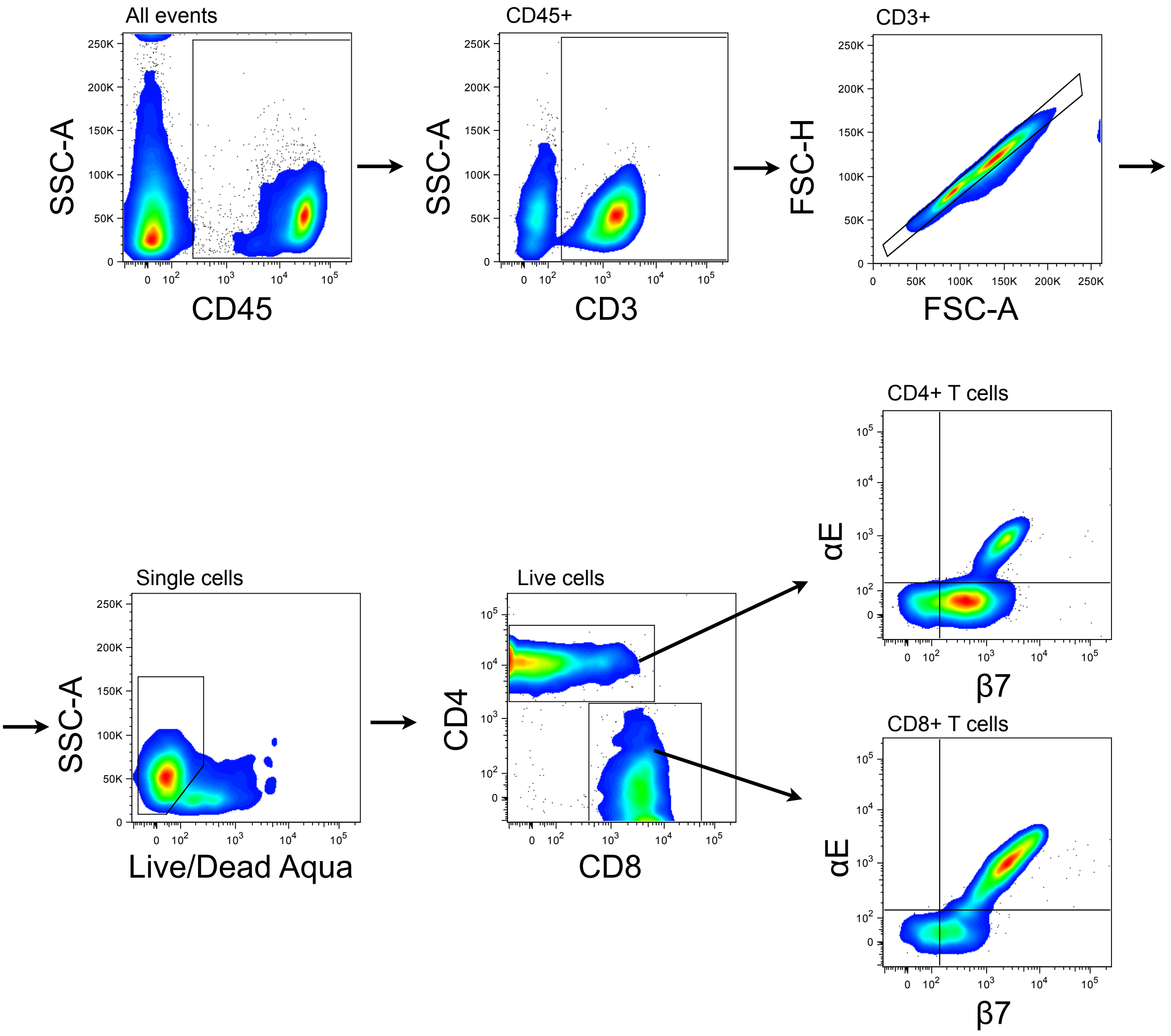

Supplementary Figure 1: Gating strategy to identify integrin expressing T cells by flow cytometry

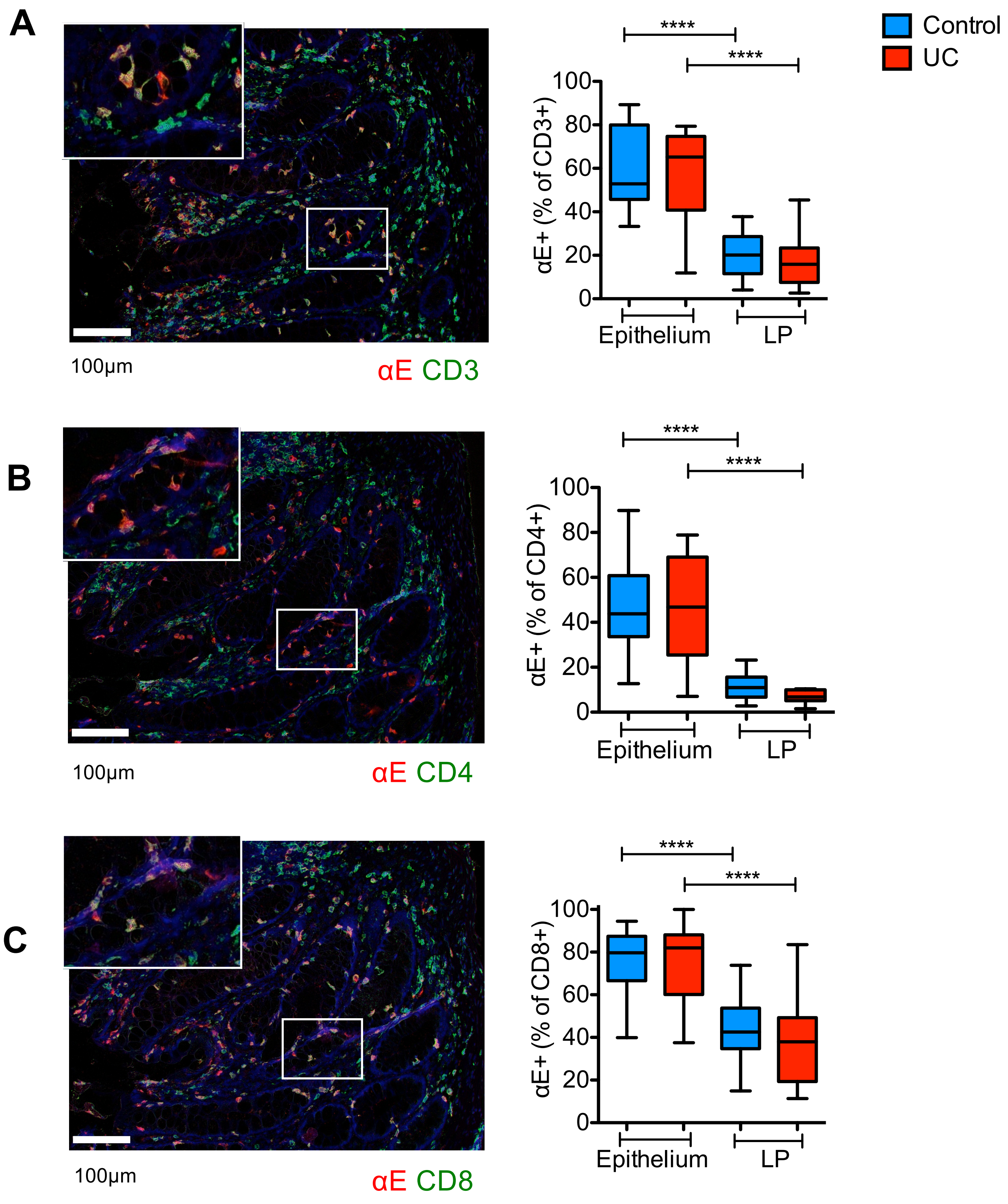

Supplementary Figure 2: A higher proportion of intraepithelial than lamina propria lymphocytes express the αE integrin

Dual stain IHC was performed on formalin-fixed paraffin-embedded colonic biopsies to evaluate co-expression of the αE integrin (shown in red) with **(A)** CD3, **(B)** CD4 and **(C)** CD8 lymphocytes (all shown in green) in colonic biopsies from control subjects (n=16) and patients with active UC (n=14). Representative Nuance spectrally unmixed images (with magnified insert) from a UC patient are shown for each pair of stains. Automated morphometric analysis was used to enumerate cellular expression of single or dual chromagen. Dual-expressing cells contain yellow. Scale bars represent 100µm.

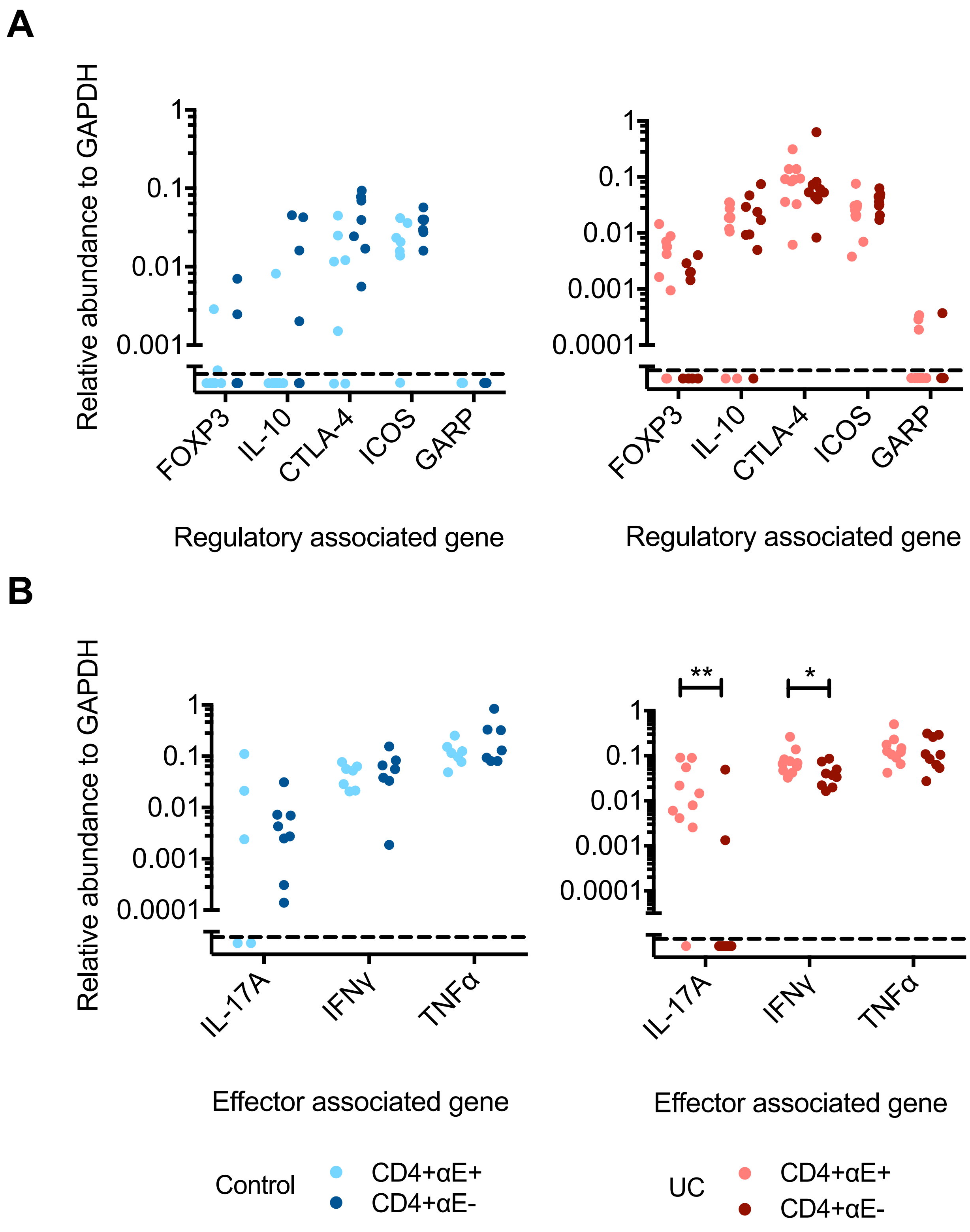

Supplementary Figure 3: CD8+αE+ and CD8+αE- colonic T lymphocytes have a similar expression of regulatory and effector genes

FACS-sorted CD8+αE+ and CD8+αE- T lymphocytes from colonic biopsies harvested from control subjects (n=8) and patients with active UC (n=10) were analysed by qPCR for expression of **(A&B)** regulatory and **(C&D)** effector associated genes.

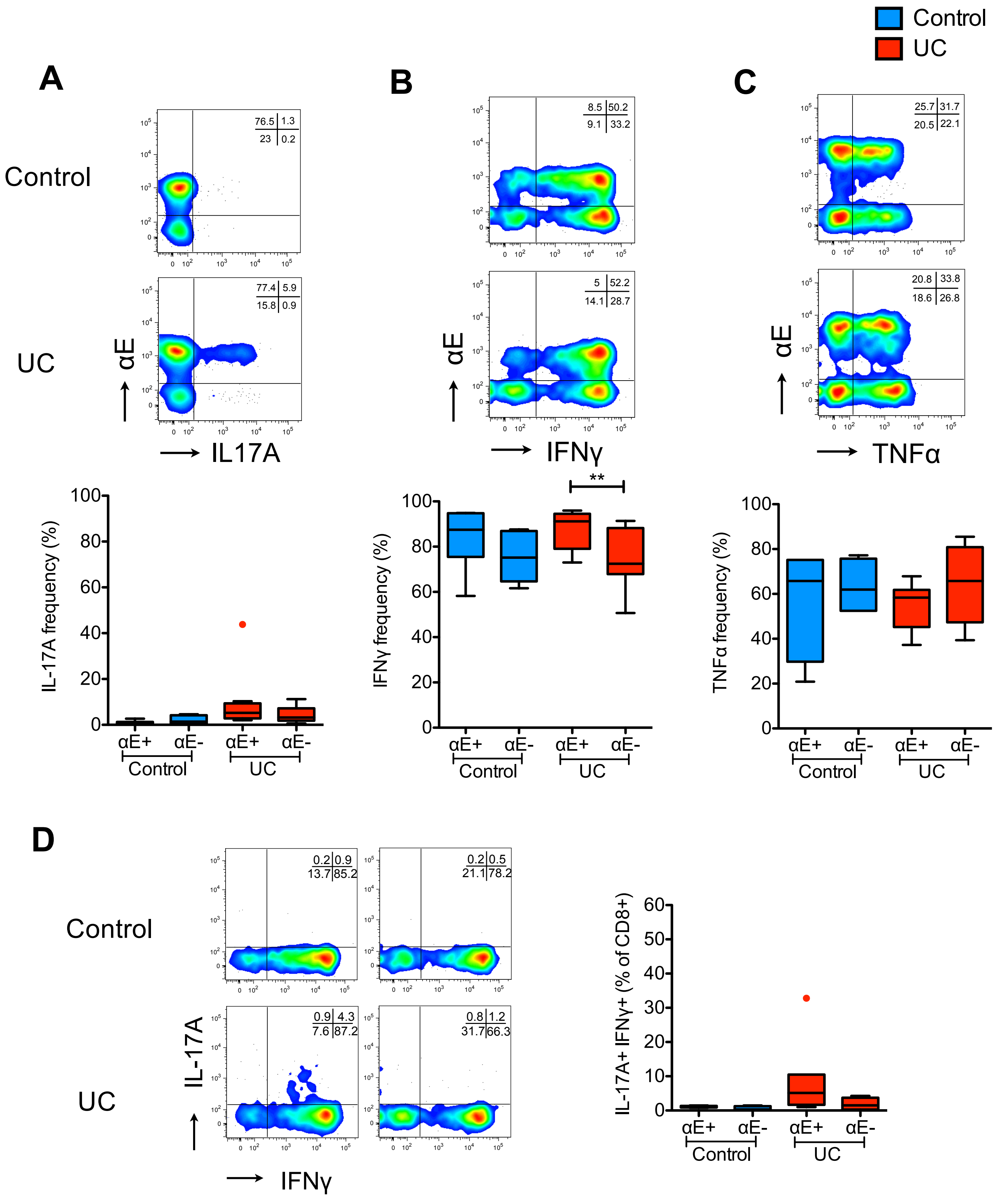

Supplementary Figure 4: Both CD8+αE+ and CD8+αE- lymphocyte subsets have a low potential to express IL-17A, but a high potential to express IFNγ and TNFα

Colonic CD8+αE+ and CD8+αE- T lymphocytes were stimulated *ex-vivo* and evaluated by FACS to determine effector cytokine production of **(A)** IL-17A (n=6 control subjects and n=8 patients with active UC), **(B)** IFNγ (n=6 control subjects and n=8 patients with active UC), **(C)** TNFα (n=4 control subjects and n=6 patients with active UC), and **(D)** dual IL-17A and IFNγ (n=4 control subjects and n=6 patients with active UC). Representative 2-colour FACS plots and summary Tukey box plots are shown for each cytokine or cytokine pair.

Antibody	Fluorochrome	Isotype	Clone	Host	Source
αE integrin	FITC	IgG1k	Ber-ACT8	Mouse	BD Pharmingen
αE integrin	PE	IgG1k	Ber-ACT8	Mouse	eBioscience
β7 integrin	PE	IgG2ak	FIB504	Rat	BD Pharmingen
α4β7 integrin	Alexa Fluor 647	IgG1k	ACT1	Mouse	From hybridoma
CD45	PE	IgG1k	2D1	Mouse	eBioscince
CD3	eFluor450	IgG2ak	OKT3	Mouse	eBioscience
CD3	PerCP Cy5.5	IgG2ak	OKT3	Mouse	eBioscience
CD4	PE-Cy7	IgG1k	RPA-T4	Mouse	eBioscience
CD8α	APC Cy7	IgG1k	HIT8a	Mouse	Biolegend
CD161	APC	IgG1k	DX12	Mouse	BD Pharmingen
IL-17A	PE	IgG1k	N49-653	Mouse	BD Pharmingen
TNFα	Alexa Fluor 647	IgG1k	Mab11	Mouse	Biolegend
IFNγ	APC	IgG1k	B27	Mouse	BD Pharmingen
Isotype	FITC	IgG1k	P3.6.2.8.1	Mouse	eBioscience
Isotype	PE	IgG1k	P3.6.2.8.1	Mouse	eBioscience
Isotype	PE	IgG2ak	R39-95	Rat	BD Pharmingen
Isotype	PE-Cy7	IgG1k	P.3.6.2.8.1	Mouse	eBioscience
Isotype	Alexa Fluor 647	IgG1k	MOPC-21	Mouse	Biolegend
Isotype	APC	IgG1k	MOPC-21	Mouse	BD Pharmingen
Isotype	APC Cy7	IgG1k	MOPC-21	Mouse	Biolegend

Supplementary Table 1: Antibodies used for cell surface and intracellular flow cytometric analysis

Antibody	Host	Clone	Dilution	Manufacturer
αE integrin	Mouse	EPR4166	1:2000	Epitomics
CD3	Mouse	LN10	1:50	Leica
CD4	Rabbit	Sp35	Pre-dilute	Ventana
CD8	Mouse	UCH-T4	1:40	Ventana
FOXP3	Mouse	259D	1:100	Biolegend
Isotype	Mouse	MOPC21	Pre-dilute	Ventana
Isotype	Rabbit	-	Pre-dilute	Ventana

Supplementary Table 2: Immunohistochemistry antibodies
